# Ex vivo model exhibits protective effects of sesamin against destruction of cartilage induced with a combination of tumor necrosis factor-alpha and oncostatin M

**DOI:** 10.1186/s12906-016-1183-0

**Published:** 2016-07-11

**Authors:** Manatsanan Khansai, Kanchanit Boonmaleerat, Peraphan Pothacharoen, Thanyaluck Phitak, Prachya Kongtawelert

**Affiliations:** Thailand Excellence Center for Tissue Engineering and Stem Cells, Department of Biochemistry, Faculty of Medicine, Chiang Mai University, Chiang Mai, 50200 Thailand

**Keywords:** Sesamin, Cartilage explant, TNF-α, OSM, Cartilage degradation, Rheumatoid arthritis

## Abstract

**Background:**

Rheumatoid arthritis (RA) is an autoimmune disease associated with chronic inflammatory arthritis. TNF-α and OSM are pro-inflammatory cytokines that play a key role in RA progression. Thus, reducing the effects of both cytokines is practical in order to relieve the progression of the disease. This current study is interested in sesamin, an active compound in sesame seeds. Sesamin has been shown to be a chondroprotective agent in osteoarthritis models. Here, we have evaluated a porcine cartilage explant as a cartilage degradation model related to RA induced by TNF-α and/or OSM in order to investigate the effects of sesamin on TNF-α and OSM in the cartilage degradation model.

**Methods:**

A porcine cartilage explant was induced with a combination of TNF-α and OSM (test group) or IL-1β and OSM (control group) followed by a co-treatment of sesamin over a long-term period (35 days). After which, the tested explants were analyzed for indications of both the remaining and the degradation aspects using glycosaminoglycan and collagen as an indicator.

**Results:**

The combination of TNF-α and OSM promoted cartilage degradation more than either TNF-α or OSM alone and was comparable with the combination of IL-1β and OSM. Sesamin could be offering protection against cartilage degradation by reducing GAGs and collagen turnover in the generated model.

**Conclusions:**

Sesamin might be a promising agent as an alternative treatment for RA patients. Furthermore, the generated model revealed itself to be an impressive test model for the analysis of phytochemical substances against the cartilage degradation model for RA. The model could be used to test for the prevention of cartilage degradation in other biological agents induced with TNF-α and OSM as well.

## Background

Rheumatoid arthritis (RA) is a systemic autoimmune disease associated with chronic inflammation of the synovial membrane (synovitis) and cartilage destruction of the affected joints [1]. The progression of RA is primarily associated with synovitis caused by a persistent immune response from the accumulation of many immune cells [2]. Consequently, the site is surrounded by a large amount of cytokines and chemokines that are produced from immune cells and inflamed synovial fibroblasts. The major pro-inflammatory cytokines found in the synovial fluid of RA patients are Tumor necrosis factor-alpha (TNF-α) and Oncostatin M (OSM) [3]. Previous studies have revealed that the elevation of TNF-α and OSM levels are implicated in the up-regulation of matrix metalloproteinase enzymes such as the MMPs and ADAMTs families in the chondrocyte cells. For instance, the degrading enzymes implicated in RA are MMP1, MMP3, MMP9, MMP13, ADAMTS4 and ADAMTS5 [4, 5]. Thus, these active enzymes are the actual cause of progressive cartilage destruction [4–6].

TNF-α is a cytokine that has the potential function of stimulating many cell types [7]. It is produced primarily by monocyte and macrophage cells, but it is also synthesized by B cells, T cells and fibroblasts [7]. The main function of TNF-α is to promote inflammation. It can act as a potent paracrine molecule by inducing other pro-inflammatory cytokine products such as interleukin-1β (IL-1β), interleukin-6 (IL-6), interleukin-8 (IL-8) and granulocyte-monocyte colony-stimulating factor (GM-CSF) [8–10]. In particular, increased amounts of these pro-inflammatory cytokine products in RA joints promote the up-regulation of degrading enzymes at the site of inflammation. The most potent enhancer of TNF-α in RA progression is the IL-6 family [4, 5].

Oncostatin M (OSM) is a pleiotropic cytokine that is produced by activated T-cells and macrophages [5]. Its structure and function are similar to the IL-6 family. This cytokine has been reported to contribute to joint inflammation in RA patients [11]. It is also known to modulate extracellular matrix turnover by controlling the balance between MMPs and the inhibitor (TIMP) at the damage site [12]. OSM also induces ADAMTS enzyme production in chondrocyte cells [6]. Additionally, OSM can act as an enhancer for IL-1β, TNF-α and interleukin-17 (IL-17) in the progression of cartilage destruction by promoting the production of degrading enzymes [5, 13].

Powerful tools are used to study the role of cytokines in inducing cartilage degradation, these include: in vitro models such as the monolayer culture model and the 3D culture model, in vivo models such as the animal model and ex vivo models such as the cartilage explant model [5, 14–16]. However, each model has its own limitations. For example, the monolayer culture model cannot explain the overall progression of the disease and the 3D culture model is suitable for anabolic study rather than a study on catabolic function [17]. The animal model seems to be the best model to study the progression of the disease and for drug development. Nevertheless, animal experiments have a serious limitation due to the differences in biological responses; this is due to the differences between species. Furthermore, the model is made even more difficult because of the complicated biological systems involved as revealed in in vivo studies [18, 19]. In order to avoid such systemic and environmental complications in the animal model, the cartilage explant culture model has been used. It is considered the most effective choice due to its ability to closely represent the physiological situation, while at the same time, it downplays any unexpected biological responses outside of the condition of interest found in in vivo studies [19].

Sesamin is a major lignan found in *Sesamum indicum* Linn. Seed [20]. It has been reported as having the properties needed to act as an anti-oxidant and anti-inflammatory compound in rat models [21]. Other evidence points to sesamin having the ability to inhibit lipopolysaccharide (LPS)-induced inflammation by suppressing the p38 mitogen-activated protein kinase (p38 MAPK) and nuclear factor-kB (NF-kB), which are the main pathways that regulate cytokine production [22]. Interestingly, sesamin has also demonstrated the fact that it is a chondroprotective agent [23]. Sesamin has shown the ability to protect against cartilage destruction in IL-1β-induced inflammation in the Osteoarthritis (OA) model, both in in vitro and in vivo studies [23]. Based on this evidence, it is also possible that sesamin could protect against cartilage degradation in other joint diseases such as rheumatoid arthritis.

The purpose of this study was to evaluate a porcine cartilage explant as a cartilage degradation model induced by TNF-α and/or OSM and to investigate the effects of sesamin on TNF-α and OSM in the porcine cartilage explant model.

## Methods

### Chemicals

Chemicals and supplements were obtained from the following suppliers: cell culture supplements such as Dulbecco’s Modified Eagle’s Medium (DMEM), Penicillin-Streptomycin and 0.25 % Trypsin EDTA were purchased from Life Technologies (Burlington, Ontario, Canada). Recombinant Human TNF-α was purchased from Peprotech (Rocky Hill, USA). Human recombinant Oncostatin M (OSM) was obtained from R&D Systems (Minneapolis, MN). Sesamin was extracted from sesame seeds (*Sesamum indicum* Linn.) and analyzed for its chemical structure by NMR/MS (MW 354.35). Silica gel column chromatography was purchased from Merck Millipore (Merck KGaA, Darmstadt, Germany). Hypersil Column chromatography for high performance liquid chromatography: Hypersil ODS- 25, 250 4.6 mm was purchased from Thermo electron corporation. Sesamin standard was obtained from Sigma Aldrich (Saint Louis, Missouri, USA). Dexamethasone was obtained from Sigma Aldrich (Saint Louis, Missouri, USA). Papain was purchased from Sigma Aldrich (Saint Louis, Missouri, USA). β- nicotinamide adenine dinucleotide reduced disodium (β-DPNH) was purchased from Sigma Aldrich (Saint Louis, Missouri, USA). Rabbit anti-human ADAMTS4 antibody and mouse anti-human MMP13 antibody were obtained from Merck Millipore (Merck KGaA, Darmstadt, Germany). Goat anti-rabbit IgG conjugated HRP and horse anti-mouse IgG conjugated HRP were obtained from Cell Signaling Technology (Danvers, MA, USA). Bradford reagent was obtained from Bio-rad (Bio-Rad Laboratories (Singapore) Pte. Ltd.). Nitrocellulose membranes were purchased from Amersham (Hybond-C Super; Amersham Pharmacia Biotech). Semi-dry blot machine was purchased from Bio-rad (Bio-Rad Laboratories (Singapore) Pte. Ltd.). SuperSignal West Femto Maximum Sensitivity Substrate kit was purchased from Thermo Sciencetific (Thermo Fisher, Lifetechnologies). Gel Documentary system was purchased from Biorad (Bio-Rad Laboratories (Singapore) Pte. Ltd.). Mouse Anti-human type ΙΙ collagen antibodies were purchased from Abcam (Cambridge, United Kingdom). Anti-mouse antibodies conjugated with HRP were obtained from Sigma Aldrich (Saint Louis, Missouri, USA). DAB reagent was purchased from Invitrogen (Thermo Fisher Scientific, Waltham, Massachusetts, USA).

### Cartilage explant preparation [24]

For the porcine cartilage explant model, metacarpo- and metatarso-phalangeal joints from pigs provided by a local slaughterhouse were dissected for articular cartilage. Cartilage discs (approximately 3 mm^2^) were randomly selected. Selected cartilage discs or explants (3 pieces per well, approximately 30 to 35 mg total) were cultured in a 24-well tissue culture plate with media without FCS (DMEM) containing 200 units/ml penicillin, 200 mg/ml streptomycin and 50 μg/ml gentamicin. The explants were maintained in a humidified incubator with 5 % CO_2_ at 37 °C overnight for sterility testing. After the sterility tests were done, the explants were maintained in a serum-free media (DMEM) for 24 h prior to the first day of treatment (this counts as day 0 for the media). Cultured media were collected on day 7 of the culture and replaced with the same treatment every seven days of the culture until the experiment ended (on day 14 or 35). The explant cultured media were stored at −20 °C until they were analyzed for indications of degradation.

### Sesamin extraction

Sesamin was prepared according to Phitak’s report [23]. Sesame seeds were amassed from Lampang, Thailand. Voucher specimens were acquired (BKF No. 138181) from the National Park, Wildlife and Plant Conservation Department, Ministry of Natural Resources and Environment, Bangkok, Thailand. For extraction, fine, air-dried powder from the sesame seeds was permeated 6 times with 4 liters of hexane for 3 days at room temperature. The liquid solution from the extraction was separated and dried under reducing pressure conditions to afford a crude hexane extract. Silica gel column chromatography system was used to separate the crude extract. Elution was started using hexane, and was progressively supplemented with ethyl acetate in hexane up to 20 % v/v. Eluent was collected as a fraction, and was then inspected by thin layer chromatography. Fractions were evaporated. Then, the dried fraction that gave colorless crystals was returned and separated by silica gel column. Afterward, the distinctive sub-fraction was purified by crystallization with ethanol to earn colorless needle crystals. Nuclear magnetic resonance spectroscopy and mass spectrometry were performed and the extract was specified as sesamin. The purified extract was verified by comparison with a trustworthy standard by co-chromatography using high performance liquid chromatography (Linear gradient system compose of Acetonitrile and H_2_O (50:50 to 70:50 v/v) at the flow rate of 1.0 ml/min. Eluent was investigated at a wavelength of 280 nm. The purified sesamin used in this study was confirmed for its structure by NMR/MS and it was found to be identical to trustworthy sesamin sources (Sigma Aldrich) by HPLC.

### Cartilage treatment [25]

Porcine cartilage explants were treated with various concentrations of sesamin in the presence of 20 ng/ml recombinant human IL-1β and 25 ng/ml recombinant human Oncostatin M (OSM) (control model) or 25 ng/ml recombinant human TNF-α and 25 ng/ml recombinant human OSM (tested model). Therapeutic control (25 nM Dexamethasone) was used in the test model only. The untreated cartilage explants were used as the control group. Throughout the 35 days of incubation, the media were collected and replaced every 7 days of cultivation. Cultured media were collected in order to analyze cartilage matrix molecules (S-GAGs and collagen release) and catabolic enzyme (ADAMTS4 and MMP13 release) induction. After 5 weeks of incubation, cartilage tissues were collected and digested with 2 units/ml papain at 60 °C, and then an assay was performed for any remaining uronic acid and collagen.

### Cytotoxicity test

The toxicity of all treatments against cells in the explant was determined by a colorimetric assay, based on the measurement of lactate dehydrogenase (LDH) activity in the culture media. The culture media from the cartilage explants treated with 30 mM H_2_O_2_ were used as a positive control_._ The cytotoxicity of each treatment against cartilage was assayed after treatment of the explants for 7, 14, 21, 28, and 35 days. The experiment was processed using Berger-Broida protocol. The reaction consisted of 375 μl of 2 mM pyruvate substrate, 625 μl of 0.3 mM β-nicotinamide adenine dinucleotide reduced disodium (β-DPNH) in phosphate buffer solution (50 mM of NaHPO_4_ and 8 mM of KH_2_PO_4_, pH 7.4), and 100 μl of the sample was incubated at 37 °C for 30 min. After that, 1 ml of the color reagent (2, 4-dinitrophenylhydrazine in 1 N HCl) was added and was allowed to stand at room temperature for 20 min. Stop solution (0.4 N NaOH) was added and the absorbance of the developing color (brown color) was measured at 450 nm. The value of the LDH released was calculated from a standard curve.

### Determination of sulfated-proteoglycans (S-GAGs) released in culture media

Sulfated GAGs (S-GAGs) released in media were identified using a dimethylmethylene blue (DMMB) assay [26]. This process was performed in both the cultured media samples and in the standard (Chondroitin 6-sulfate (CS-C)). The DMMB solution was added to the diluted samples, standard and blank solution (200 μl: 50 μl) prior to measuring absorbance at 620 nm. The value of the S-GAGs released was calculated from a standard curve.

The percentage of the S-GAGs released from the cartilage explants that were induced with TNF-α and OSM was calculated as follows:

$$ \% change=\frac{\left( value\  from\ D7\  or\ D14\  or\ D21\  or\ D28\  or\ D35\  medium- value\  from\ D0\  medium\right)}{value\  from\ D0\  medium}x\kern0.5em 100 $$

### Uronic acid (UA) assay for GAGs remaining in cartilage

The uronic acid levels remaining in the explants were measured after papain digestion of the cartilage discs. Measurements were taken using m-hydroxydiphenyl in a colorimetric assay [27]. Glucuronic acid lactone was used as the standard. Concentrated sulfuric acid-borate reagent (300 μl) was then added to both the standard and the samples. The reactions were incubated at 100 °C for 15 min and then cooled down on ice. The carbazole solution (50 mg carbazole in 40 ml EtOH) (12.5 μl) was added and the reactions were incubated at 100 °C for 15 min. After that, the uronic acid reaction was cooled down on ice. The absorbance of the pink color was detected using a spectrophotometer at 540 nm.

### Measurement of hydroxyproline released in media and remaining in cartilage [28]

The hydroxyproline in the samples was oxidized into a pyrrole with chloramin T at pH 6. At this intermediate stage, samples were dyed pink using 4-dimethylaminobenzaldehyde. Papain-digested cartilage and cultured media were hydrolyzed with 6 N HCl at 110°C for 24 h. After hydrolyzation, samples were freeze-dried and resolubilized with distilled water. The diluents solution (67 % propan-2-ol) and oxidant solution (50 mM chloramine T) were added to the samples, followed by a color reagent of 7.5 % dimethylaminobenzaldehyde in propan-2-ol. The reaction occurred at 60 °C for 45 min. An absorbance of peach color was measured at 540 nm.

The percentage of collagen released from the cartilage explants that were induced with TNF-α and OSM was calculated as follows:

$$ \% change=\frac{\left( value\  from\ D7\  or\ D14\  or\ D21\  or\ D28\  or\ D35\  medium- value\  from\ D0\  medium\right)}{value\  from\ D0\  medium}x\kern0.5em 100 $$

### Investigation of ADAMTS4 and MMP13 released in culture medium by Western blot analysis

The culture medium from cartilage explant D7, D14, D21, D28, and D35 were determined the protein concentration by using Bradford protein assay and protein concentration was calculated to produce equal amounts before loading in SDS-PAGE (13 % separating gel, 5 % stacking gel). The samples were separated electrophoretically and transferred to nitrocellulose membrane by semi-dry blot method. After blotting, membranes were blocked with 3 % (W/V) BSA in Tris-buffered saline with 0.1 % Tween 20 (TBS-T) for 1 h, at room temperature. After which, the membranes were washed with TBS-T prior to being incubated with primary antibody against anti-ADAMTS4 or anti-MMP13 antibody (1:500 for anti-ADAMTS4 and 1:1000 for anti MMP13 in TBS-T) overnight at 4 °C. After that, membranes were washed 5 times for 5 min with TBT-T prior to being incubated with the secondary antibody conjugated with horseradish peroxidase (1:1000 in TBS-T, anti-rabbit IgG for anti ADAMTS4 and anti-mouse IgG antibody for anti-MMP13) for 1 h at room temperature. Blots were washed 5 times for 5 min with TBS-T before being visualized using SuperSignal West Femto Maximum Sensitivity Substrate kit for enhanced chemiluminescence. The visualized results were taken using the Gel Documentary system.

### Safranin O staining

The sections were stained with safranin O to determine the amount of S-GAGs remaining in the cartilage [29]. The sections were deparaffinized using xylene and were then dehydrated using 95 % ethanol and absolute ethanol, respectively. The dehydrated sections were stained with safranin O for 30 min. After that, the stained sections were rehydrated and mounted.

### Immumohistochemistry for type II collagen

For the immunohistological analysis of the remaining type II collagen [30], after deparaffinization and rehydration, the sections were first digested with 0.25 % trypsin, then, 1.45 IU/ml testicular hyaluronidase and finally, 0.25 IU/ml chondroitinase ABC for 15 min at 37 °C for each step. Consequently, endogenous peroxidase was blocked in the sections using 3 % H_2_O_2_ in PBS and non-specific sites were blocked with 3 % BSA in PBS. Sections were incubated with mouse monoclonal anti- type II collagen (1:100) for 24 h at 4 °C. Following that, the anti-mouse polyvalent immunoglobulin conjugate HRP (1:25) was incubated for 1 h at room temperature. After being washed with PBS, the sections were color-stained using peroxidase substrate (DAB reagent set), followed by rehydration. The sections were mounted with a mounting medium and left at room temperature until they were dried.

### Statistics analyses

The significance of the differences between the groups of data was assessed using One-way ANOVA, SPSS and all groups were expressed as mean ± SD. Statistical significance was assumed at *P* < *0.05.*

## Results

### TNF-α combined with OSM promoted a high degree of cartilage degradation in the porcine cartilage explant model

**Fig. 1:**
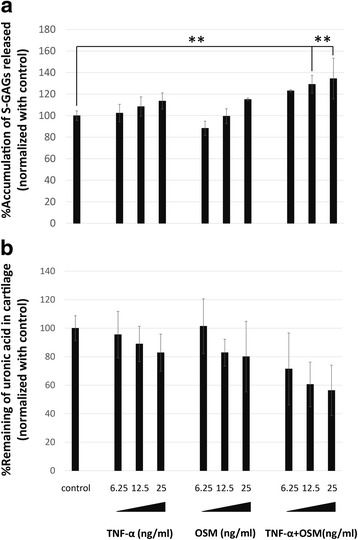
Significant S-GAGs depletion and uronic acid remaining in cartilage when induced with TNF-α/OSM alone or TNF-α in combination with OSM. The levels of S-GAGs released in the media were assayed as described in the Methods section. **a** S-GAGs released in the media represented as the percentage of accumulation of S-GAGs that were released at 7 days and 14 days. **b** Percentage of uronic acid retained in the cartilage after treatment ended (14 days). Values are presented as mean ± SD (*n* = 3). * = *p* < 0.05; ** = *p* < 0.01 versus normal control. Data represents 3 separate cartilage samples

To assess the strength of pro-inflammatory cytokines on cartilage degradation, the explants were stimulated with TNF-α or OSM alone or a combination of TNF-α and OSM at concentrations of 6.25, 12.5, and 25 ng/ml for 14 days. The cultured media were collected every 7 days and replaced with the same conditional treatment. After the experiment ended, the explants were harvested. The cultured media that were collected on day 7 and day 14 of the culture were inspected to determine S-GAGs levels, while the explants were checked for remaining amounts of uronic acid. After the 14-day period, it was shown that the presence of TNF-α or OSM alone in the porcine cartilage explant culture induced a low level of proteoglycan depletion in a dose-dependent manner (Fig. 1). TNF-α or OSM alone at 25 ng/ml induced S-GAGs release at about 113 and 115 % when normalized with the control (control = 100 %), respectively (Fig.1a). However, a combination of both cytokines increased S-GAGs release to about 134 % (Fig. 1a). Moreover, it was shown in the levels of remaining uronic acid that OSM had a synergistic effect with TNF-α (Fig. 1b). The combination of 25 ng/ml TNF-α and OSM exhibited the highest level of the cartilage degradation marker. This concentration was chosen selected for use in further experiments.

### TNF-α and OSM induced S-GAGs degradation that was comparable with IL-1β and OSM induction, and sesamin showed protective effects on S-GAGs degradation with TNF-α and OSM

**Fig. 2:**
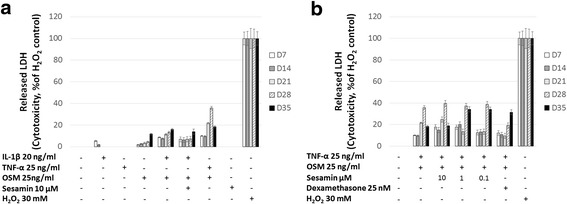
The release of LDH in the culture media. The amount of LDH released in the culture medium was determined as described in the research methodology. **a**. Percentage of LDH released in the media from the cytokine(s)-induced condition normalized against the percentage of LDH released from H_2_O_2_ treatment. **b**. Percentage of LDH released in the media from a combination of TNF-α and OSM induced-condition with sesamin normalized against the percentage of LDH released from the H_2_O_2_ treatment. Values are presented as mean ± SD (*n* = 3). Data represents 3 separate cartilage samples

Firstly, to evaluate the cytotoxicity of all conditions, the culture media from all conditions or H_2_O_2_ treatment condition were used to test LDH activity. The toxic control showed increased levels of LDH released in the media, while TNF-α, OSM, IL-1β or its combination and sesamin treatment did not appear to affect the amount of LDH released in the cultured media (Fig. 2a). Some treatments showed slightly increased levels of released LDH (Fig. 2). However, these released LDH levels did not reach toxic levels when compared with the toxic control (Fig. 2). The results indicated a nontoxicity of all treatments on chondrocyte cells.

**Fig. 3:**
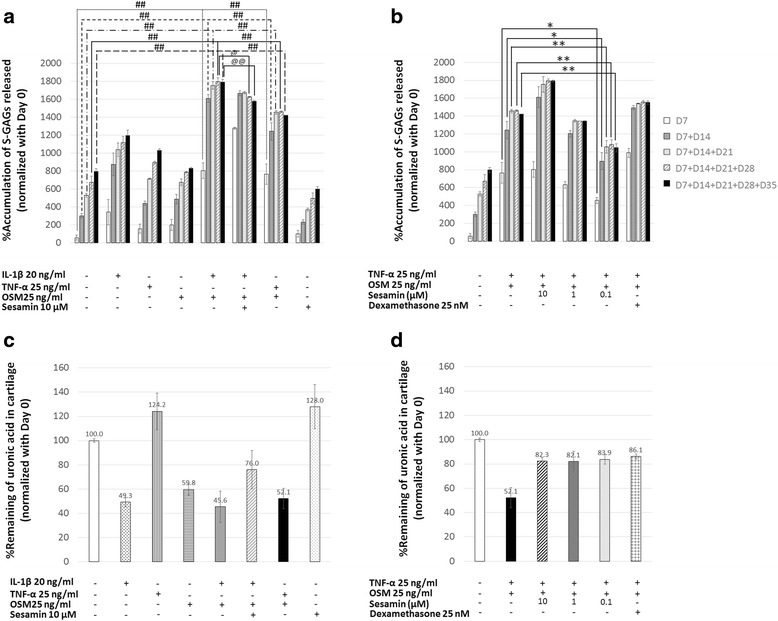
The percentage accumulation of S-GAGs released in the culture media and the percentage of uronic acid remaining in the cartilage explants versus the normal control. **a**, **b** The percentage of S-GAGs released that was accumulated and normalized against the percentage of S-GAGs released on day 0. **c**, **d** The percentage of uronic acid remaining in the cartilage after the end of the experiment. Values are presented as mean ± SD (*n* = 3). #, @, * = *p* < 0.05; ##, @@, ** = *p* < 0.01 versus control (#) or the combination of IL-1β and OSM treatment (@) or the combination of TNF-α and OSM treatment (*). Data represents 3 separate explant samples

The determination of S-GAGs degradation represented the different pattern of single-cytokine treatment and co-cytokine treatment in the porcine cartilage explant model. In IL-1β, TNF-α, and OSM alone treatment conditions, S-GAGs were continuously depleted, meanwhile both combinations of cytokine treatment conditions showed short and strong effects (Fig. 3a). The combination of TNF-α and OSM at a concentration of 25 ng/ml enhanced the depletion of GAGs in the porcine cartilage culture when compared with the TNF-α or OSM treatment alone (Fig. 3a). However, S-GAGs damaged from the combination of TNF-α and OSM condition was still lesser than the combination of IL-1β and OSM treatment (Fig. 3a). The highest level of S-GAGs released was detected in the second week of treatment and decreased by the next week. In accordance with previous reports, co-treatment with sesamin at a concentration of 10 μM exhibited a significantly lower percentage of the accumulation of S-GAGs released when compared with the IL-1β and OSM co-treatment groups (Fig. 3a). Interestingly, TNF-α and OSM co-treatment with sesamin at concentration 0.1 μM also decreased the percentage of S-GAGs released when compared with the cytokines-induced group. However, co-treatment with sesamin at a concentration of 10 μM showed a higher percentage of S-GAGs released (Fig. 3b). The dexamethasone co-treatment, which is currently used as a therapeutic drug, induced a higher percentage of the S-GAGs released in the second week of treatment when compared with the TNF-α and OSM co-treatment groups (Fig. 3b).

Almost all cytokine treatment conditions resulted in a reduction of uronic acid remaining in the cartilage except for the TNF-α alone condition (Fig. 3c). Similarly to the S-GAGs released results, the combination treatment of TNF-α and OSM could reduce the percentage of uronic acid remaining at the same level as the co-treatment of IL-1β and OSM (Fig. 3c). Sesamin was able to slightly retain uronic acid remaining in the cartilage under the stimulating condition that was likely expressed in the dexamethasone treatment (Fig. 3b, d).

### TNF-α and OSM induced collagen degradation and sesamin showed protective effect against these cytokines

**Fig. 4:**
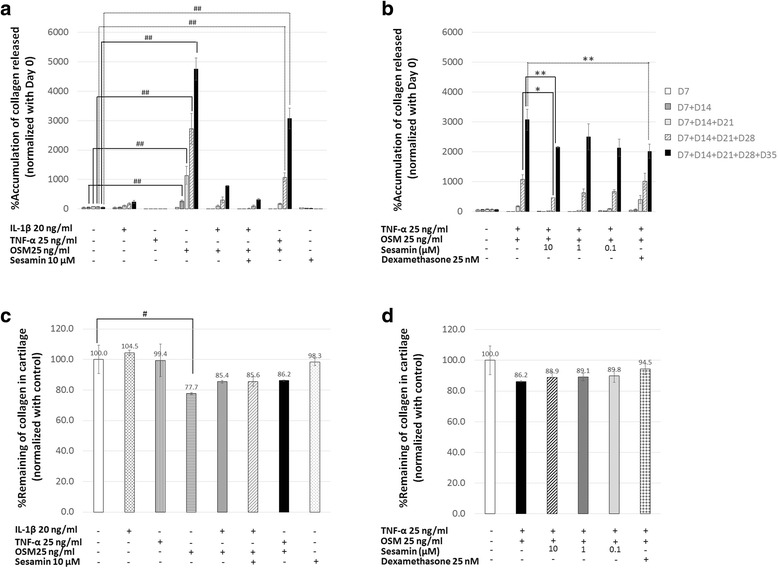
The percentage of collagen released in accumulation in the media and the percentage of collagen that remained in the cartilage explants under different conditions. The amount of collagen released in the media and the amount of collagen remaining in the digested cartilage were determined as described in the Methods section. **a**, **b** The change in percentage of collagen released that was accumulated in the media under various conditions and normalized against the percentage of collagen released on day 0. **c**, **d** The percentage of collagen that remained in the cartilage explants after the end of the experiment. Values are presented as mean ± SD (*n* = 3). #, * = *p* < 0.05; ##, ** = *p* < 0.01 versus control (#) or versus the combination of TNF-α and OSM treatment (*). Data represents 3 separate explant samples

The change of collagen in the cartilage was investigated for both its degrading and remaining aspects. The degradation of collagen was determined by measuring its release in the culture medium, and the remaining aspect was studied by quantifying the amount of collagen existing in the explant after the culture period had ended. As expected, the level of collagen released from cartilage was detected at high levels near the end of the experiment’s duration (Fig. 4a, b). From these results, elevated collagen degradation levels were shown in the cartilage, which was cultured under the induced condition (Fig. 4a). Therefore, co-treatment of TNF-α and OSM significantly induced high levels of collagen depletion, while the induction level was lower than that which was induced by OSM alone (Fig. 4a). While IL-1β combination with OSM showed a slight increase in the amount of collagen released in the culture media (Fig. 4a). A co-treatment with sesamin relieved those effects in both the TNF-α/OSM and IL-1β/OSM induced conditions (Fig. 4a, b). A significant reduction in the percentage of collagen released in the medium was found when sesamin was introduced at a concentration of 10 μM (Fig. 4b). Additionally, the collagen remaining in the cartilage produced harmonious results; the collagen remaining in the explants decreased when stimulated with pro-inflammatory cytokines, while in the presence of the sesamin treatment, sesamin proved to lighten the unwanted effects (Fig. 4c, d). The sesamin treatment’s results were comparable to those of the dexamethasone treatment (Fig. 4b, d).

### TNF-α and OSM induced the releasing of ADAMTS4 and MMP13 that was comparable to IL-1β and OSM induction

**Fig. 5:**
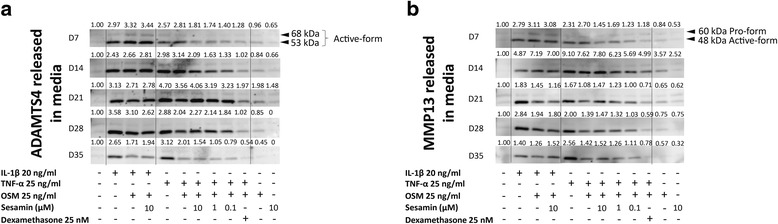
Western Blotting results for mornitoring the changes of ADAMTS4 and MMP13. The releasing of ADAMTS4 and MMP13 in the media were investigated as described in the Methods section. **a** The change in ADAMTS4 released in the media. **b** The change in MMP13 released in the media. The number above each band indicates band density (total density = active form + proform)

The matrix metalloproteinase enzymes that were involved in aggrecan and collagen degradation were also observed. In this experiment, ADAMTS4 and MMP13 expression levels were determined in all conditioned media. From these results, both IL-1β and TNF-α alone treatments induced matrix metalloproteinase enzymes released while OSM treatment alone revealed only slight effects (Fig. 5). The combination cytokines system showed a stronger induction level when compared to the single-cytokine system. Moreover, TNF-α and OSM induced ADAMTS4 and MMP13 were released at the same levels as the IL-1β in combination with OSM. In the IL-1β and OSM treatment, 10 μM of sesamin could not inhibit the cytokine effect in terms of both ADAMTS4 and MMP13 expression. However, sesamin demonstrated the slightly reduced effects on both ADAMTS4 and MMP13 production in the TNF-α and OSM condition (Fig. 5).

### TNF-α combination with OSM demonstrated severe cartilage degradation and sesamin could protect GAGs and collagen depletion from cartilage induced with TNF-α and OSM

**Fig. 6:**
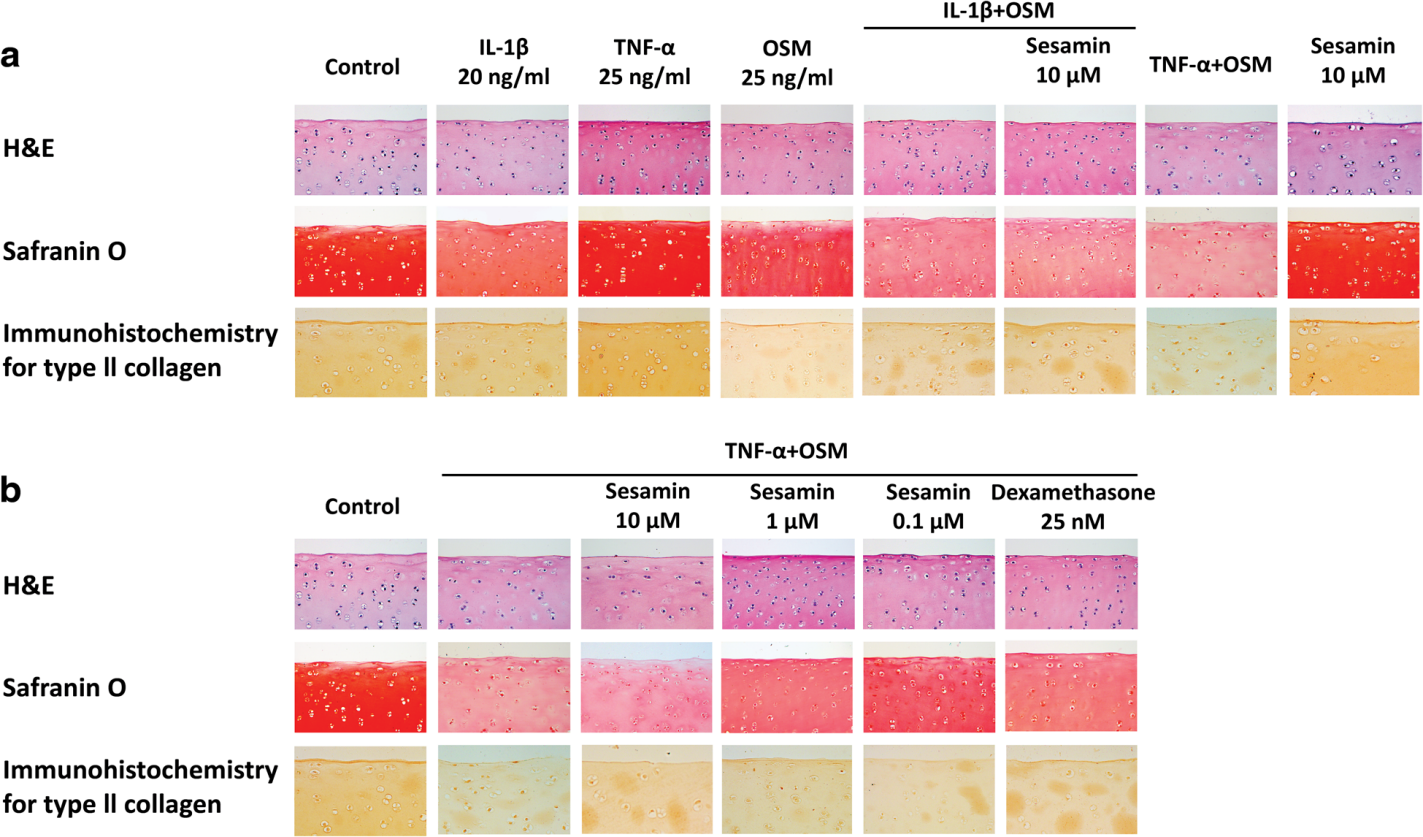
Histology staining; H&E staining for chondrocyte cell morphology, Safranin O staining for S-GAGs in cartilage (red color) and immunohistochemistry staining for type ΙΙ collagen remaining in the cartilage (brown color) (x400). **a** Histology staining of cytokine(s)-induced conditions. **b** Histology staining of co-treatment of TNF-α and OSM induced-condition with sesamin

The important evidence that supported the chondroprotective effects of sesamin against TNF-α/OSM-induced cartilage degradation came from a histological examination (Fig. 6a). TNF-α/OSM and IL-1β/OSM caused severe cartilage damage when compared with other conditions, which was observed due to the cartilage’s abnormal appearance (Fig. 6a). Additionally, the chondrocytes seemed unhealthy when compared with the normal specimens in the control group. Moreover, the surface of the cartilage explants when under the induced condition seemed decomposed. However, the presence of sesamin in the induction system resulted in better chondrocyte cell morphology and a healthy appearance of the cartilage, which was similar to results found in the dexamethasone treatment (Fig. 6b). Furthermore, the results clearly indicated that the induction with TNF-α in combination with OSM caused a severe loss of S-GAGs in the cartilage as same as IL-1β and OSM. The presence of sesamin could protect S-GAGs from depletion in both combinations of cytokine-induced cartilage. Although, in the TNF-α/OSM treatment, the lower concentration value of sesamin seemed to maintain S-GAGs more effectively than was found with the higher concentration value of sesamin (Fig. 6b). For type ΙΙ collagen content, the long-term induction of cartilage with TNF-α and OSM caused a severe loss of collagen (Fig. 6). Notably, collagen that was co-cultured with sesamin was protected from depletion when sesamin was introduced in a dose-dependent manner (Fig. 6b). However, the protective effects of sesamin were shown to be less than those of the dexamethasone treatment.

## Discussion

Rheumatoid arthritis (RA) is a chronic systemic autoimmune disease. It is characterized by chronic inflammation of the synovial membrane (synovitis) and progressive cartilage damage [1]. The progression of RA is primarily associated with synovitis caused by a persistent immune response from the accumulation of many immune cells [2]. Consequently, the site is surrounded by a large amount of cytokines, chemokines and proteolytic enzymes, which affect the cartilage and bone structure [2]. Cartilage destruction in RA is caused by a loss of extracellular matrix such as proteoglycans and collagen that are controlled by matrix metalloproteinase enzymes (MMPs) and a disintegrin and metalloproteinase with thrombospondin motifs (ADAMTS) synthesized from chondrocyte cells [4, 5]. These enzymes are up-regulated by a variety of cytokines, especially TNF-α and OSM that emerge during an inflammatory event [7]. These cytokines are also known as pro-inflammatory cytokines and are closely involved in RA [3]. In this study, the cartilage explant model was used to observe cartilage degradation. Even though the cartilage explant model presents numerous benefits, this method also has its own limitations [31]. Some limitations of the cartilage explant model could not be avoided. When employing this method, the amount of chondrocyte cells in the explant in each treatment could not be controlled, even when utilizing measurement by weight. The cutting of the cartilage also causes the chondrocyte cells to die. In order to meet the demand for the experiment, cartilage samples were obtained from various sources. It is quite difficult to control the response from individual samples. However, this model more closely represents the physiological environment than does the monolayer culture model and this model minimizes the presence of the complicated systems found in the animal model study [18, 19]. The experiment of this study was designed to mimic the microenvironment surrounding the inflammatory joint found in RA. The designed experiment was also investigated compared with IL-1β and OSM, which is a well-known inducer for the cartilage explant model. Throughout the experiment, the changes in the samples’ proteoglycans (GAGs), collagen, aggrecanase (ADAMTS4), and collagenase (MMP13) were monitored. The breakdown of S-GAGs and collagen from the cartilage is an approved method of marking cartilage degradation. S-GAGs and collagen depletion leads to a malfunction of the extracellular matrix and generates cartilage dysfunction [32, 33]. It is believed that the loss of GAGs, especially aggrecan, is the primary step in the process of cartilage destruction [33]. In this study, the depletion of S-GAGs was investigated by monitoring the levels of S-GAGs released in the media. This is a sign of the degeneration of the damaged cartilage. Uronic acid remaining in the cartilage was also observed, as this is related to the number of remaining GAGs. Moreover, changes in the collagen were monitored by observation of the collagen that was released in the cultured media. The release of collagen indicates the presence of severely damaged cartilage because the loss of the fibrillar structure is a cause of irreversible tissue destruction [34]. The collagen remaining in the explants is considered to be retained collagen. Furthermore, representative of the matrix metalloproteinases enzymes, namely ADAMTS4 and MMP13, which function as aggrecanase and collagenase, respectively, were also investigated. Both enzyme production levels reveal the role of cytokine-induced cartilage damage [6, 35]. A histological staining was performed in order to confirm the amounts of S-GAGs and collagen retained.

In this study, it was found that only IL-1β or TNF-α or OSM-treated cartilage explants caused a slight depletion of S-GAGs (an amount harmonious with the level of uronic acid remaining in the explants). In addition, TNF-α combined with OSM was found to have additive effects when used as a treatment in the cartilage explants. These results were in accordance to those of the co-treatment of IL-1β and OSM. The results suggest that the combined TNF-α and OSM treatment is appropriate for use in the cartilage explant model for the study of cartilage degradation. In fact, the combination treatment is closer to the actual pathogenic situation than the single-cytokine systems. This study has shown that long-term exposure of TNF-α and OSM around the porcine cartilage explant model can cause severe cartilage damage. From these results, it has been demonstrated that S-GAGs depletion occurs in the early stages of degradation, while the breakdown of collagen occurs in the late stages. S-GAGs depletion was definitely detected during the second week of cytokine induction and it continued to be present until the end of the experiment (the fifth week). This suggests that S-GAGs breakdown can be used as an early biomarker of cartilage destruction.

As for collagen depletion, only IL-1β or TNF-α treated cartilage explant produced a harmless effect to collagen, whereas only the OSM treated cartilage explant showed a highly degraded level of collagen. There is no other evidence to support the depletion of collagen from the treatment with OSM alone. However, in the actual situation, the rheumatic joint is surrounded by many cytokines. For this reason, the combined cytokine system is more similar to the pathophysiology of the disease than the single-cytokine system. Therefore, this study has proven that cartilage damage that is induced by a combination of TNF-α and OSM leads to significant collagen breakdown (this occurred in the later periods of the experiment), whereas a combination of IL-1β and OSM had a lighter effect on collagen degradation. Thus, the TNF-α and OSM co-treatment model is suitable for the study of a cytokine induced cartilage degradation disease, as IL-1β combined with OSM. Moreover, collagen breakdown can also be used as a marker for severe cartilage degradation.

The mechanism of cytokine induced S-GAGs and collagen depletion in RA lesions is caused by the overproduction of matrix metalloproteinase enzymes [3–5, 7]. ADAMTS4 and MMP13 proteins were chosen as a representative of the enzyme group because there is a significant amount of evidence to suggest that both enzymes are regulated by TNF-α, IL-1β or OSM [5, 34, 36]. From these studies, the co-cytokine treatment system gave a different result from the single-cytokine system. IL-1β or TNF-α treatment showed a prolonged level of induction for both enzymes when TNF-α or IL-1β in combination with OSM presented a short and strong level of induction. OSM alone slightly induced the release of both ADAMTS4 and MMP13. This result is inconsistent with the results of the collagen released, for which OSM showed the strongest effect. OSM might induce some other matrix metalloproteinase enzymes except ADAMTS4 and MMP13. TNF-α or IL-1β combination with OSM strongly induced ADAMTS4 and MMP13 that were released within a 2-week period and decreased the induction by the next week. Moreover, this inconsistency might be due to the multi-properties of OSM. OSM has been acknowledged for its pro- and anti-inflammatory properties [37].

Sesamin is an active compound from sesame seeds that has been identified as an agent that has the dominant property of being an anti-inflammatory substance [20, 21]. It has also been proven to be a chondroprotective agent in the osteoarthritis model induced with IL-1β [23]. As a consequence of a number of previous experiments, this study tested the properties of sesamin by investigating its ability to protect against cartilage damage. This was achieved by using the porcine cartilage explants induced with a combination of IL-1β or TNF-α with the OSM model. The use of these models had demonstrated that sesamin did show protective effects. Because of the presence of sesamin, there was a reduction in the amounts of both early (S-GAGs) and late (collagen) cartilage degradation markers in the induced cartilage. The release of S-GAGs and collagen was significantly reduced when compared with the release found in the cytokine control group. The effects of sesamin were also found in the direct measurements of both S-GAGs and collagen in the cartilage during a histological examination. Sesamin also slightly reduced ADAMTS4 and MMP13 enzymes that were released in the early stages of these models. The greatest concentration of sesamin that was found to protect cartilage from TNF-α and OSM induction in this study was 0.1 μM. In addition, dexamethasone, which is a medicine currently used for the treatment of cartilage degradation, was included in this investigation. Previously, dexamethasone has been extensively reported on concerning its ability to up-regulate Interleukin-10 (IL-10), which is a cytokine that induces the synthesis of endogenous TNF-α inhibitors, such as soluble TNF-α receptors. This cytokine has also been acknowledged to down-regulate the TNF receptor expression in immune cells [38, 39]. In this way, dexamethasone could be used as a positive control. In the experiment, the dexamethasone treatment clearly showed protective effects on cytokine-induced cartilage explants, while sesamin produced the same results that dexamethasone did. Interestingly, the effective dose of sesamin was found in the low concentration.

## Conclusions

Again, it must be stated that this study strongly confirmed that the cartilage explant model induced with TNF-α and OSM is a suitable model for the study of the effects of cartilage-protective substances. While using this appropriate model, it was found that sesamin, which is a natural substance extracted from sesame seeds, has clear chondroprotective effects. Importantly, sesamin is non-toxic to chondrocyte cells. These results suggest that sesamin has the valuable property of being able to relieve cartilage degradation events in a pathogenesis caused by the elevation of TNF-α and OSM levels. An example of such an event is RA. However, the mechanism which sesamin uses against the destruction caused by the combination of TNF-α and OSM is still unknown and further studies should be performed in order to identify this mechanism.

## Abbreviations

ADAMTS4, A disintegrin and metalloproteinase with thrombospondin motifs 4; ADAMTS5, A disintegrin and metalloproteinase with thrombospondin motifs 5; BSA, bovine serum albumin; EtOH, ethanol; GAGs, Glycosaminoglycans; GM-CSF, granulocyte-monocyte colony-stimulating factor; HPLC, high performance liquid chromatography; HRP, horseradish peroxidase; IL-17, Interleukin-17; IL-1β, Interleukin-1 beta; IL-6, Interleukin-6; IL-8, interleukin-8; LDH, lactate dehydrogenase; LPS, lipopolysaccharide; MMP1, matrix metalloprotinase 1; MMP13, matrix metalloprotinase 13; MMP3, matrix metalloprotinase 3; MMP9, matrix metalloproteinase 9; MMPs, matrix metalloproteinases; NF-kB, nucear factor-kB; NMR/MS, nuclear magnetic resonance spectroscopy and mass spectrometry; OA, osteoarthritis; OSM, oncostatin M; p38 MAPK, p38 mitogen-activated protein kinase; RA, rheumatoid arthritis; SDS-PAGE, sodium dodecyl sulfate-polyacrylamide gel electrophoresis; S-GAGs, sulfated- glycosaminoglycans; TBS, tris-buffered saline; TBS-T, tris-buffered saline with 0.1% Tween 20; TIMP, tissue inhibitor of metalloproteinases; TNF-α, tumor necrosis factor-alpha
